# The Impact of Infectious Complications after Esophagectomy for Esophageal Cancer on Cancer Prognosis and Treatment Strategy

**DOI:** 10.3390/jcm10194614

**Published:** 2021-10-08

**Authors:** Eisuke Booka, Hirotoshi Kikuchi, Yoshihiro Hiramatsu, Hiroya Takeuchi

**Affiliations:** 1 Department of Surgery, Hamamatsu University School of Medicine, 1-20-1 Handayama, Higashi-ku, Hamamatsu 431-3192, Shizuoka, Japan; booka@hama-med.ac.jp (E.B.); kikuchih@hama-med.ac.jp (H.K.); hiramatu@hama-med.ac.jp (Y.H.); 2 Department of Perioperative Functioning Care and Support, Hamamatsu University School of Medicine, 1-20-1 Handayama, Higashi-ku, Hamamatsu 431-3192, Shizuoka, Japan

**Keywords:** postoperative complication, esophageal cancer, esophagectomy, CXCL8, CXCR2

## Abstract

Despite advances in the perioperative management of esophagectomy, it is still a highly invasive procedure for esophageal cancer and is associated with severe postoperative complications. The two major postoperative infectious complications after esophagectomy are pulmonary complications and anastomotic leakage. We previously reported that postoperative infectious complications after esophagectomy adversely affect long-term survival significantly in a single institution and meta-analysis. Additionally, we reviewed the mechanisms of proinflammatory cytokines, such as C-X-C motif ligand 8 (CXCL8) and its cognate receptor, C-X-C chemokine receptor 2 (CXCR2), in contributing to tumorigenesis and tumor progression. Moreover, we previously reported that introducing minimally invasive esophagectomy, including robot assistance, laparoscopic gastric mobilization, and multidisciplinary team management, significantly reduced postoperative infectious complications after esophagectomy. Further, this review also suggests future treatment strategies for esophageal cancer, considering the adverse effect of postoperative infectious complications after esophagectomy.

## 1. Introduction

Esophageal cancer is the sixth leading cause of cancer-related mortality globally because of its high malignant potential and poor prognosis [1]. The postoperative 5-year survival rate in patients with American Joint Committee on Cancer stage I esophageal cancer is approximately 90%. This rate decreases to 45%, 20%, and 10% in patients with stages II, III, and IV diseases, respectively [2]. Esophagectomy is still the most effective treatment option, although chemoradiotherapy may be effective in treating esophageal cancer treatment [3]. Despite developments in extended lymph node dissection and perioperative management of esophagectomy, it remains a highly invasive procedure associated with severe postoperative complications [4]. The Japanese national database, including 5354 esophagectomy patients in 713 hospitals in 2011, indicated an overall morbidity rate of 41.9% and a 30-day and surgery-related mortality of 1.2% and 3.4%, respectively [5].

The effect of postoperative complications on long-term survival has been investigated in many cancers [4,6], including a recent meta-analysis of colorectal cancer studies [7]. Some reports have shown the adverse effect of postoperative esophagectomy complications on long-term survival [4,8], whereas others have reported that postoperative esophagectomy complications did not affect long-term survival [9]. We previously conducted a meta-analysis to investigate the effect of postoperative complications after esophagectomy on long-term survival [10].

The two major postoperative infectious complications after esophagectomy are pulmonary complications and anastomotic leakage [11]. This study investigated the relationship between postoperative infectious complications and cancer prognosis, explored the causes, and reviewed future treatment strategies.

## 2. The Effect of Postoperative Complications after Esophagectomy for Cancer on Survival

### 2.1. Pulmonary Complications

Using information recorded between 2011 and 2012 from a nationwide database in Japan, we reported that the rate of pulmonary complications after esophagectomy was 14.8% (1419/9584) [5]. Additionally, Ancona et al., reported that postoperative pulmonary complications (25.2%, 110/437) after esophagectomy did not affect long-term survival [12]. However, Baba et al., and Saeki et al., recently reported postoperative pulmonary complications (19.7%, 99/502 and 10.2%, 59/580, respectively) after esophagectomy had a significant negative effect on long-term survival [13,14].

We previously reported that, within a single institution, postoperative pneumonia after esophagectomy (22.5%, 64/284) had a significant negative effect on overall survival (OS) (*p* = 0.035). Furthermore, multivariate analysis revealed that the presence of pneumonia was predictive of poorer OS; the multivariate hazard ratio (HR) was 1.456 (95% confidence interval (CI) 1.020–2.079, *p* = 0.039)^4^. Furthermore, we analyzed the data from a randomized controlled trial (JCOG9907 trial); the OS of patients with pneumonia (14.5%, 22/152) was shorter than that of patients without pneumonia (HR: 1.82, 95% CI: 1.01–3.29), and progression-free survival (PFS) tended to be shorter in patients with pneumonia (HR: 1.50, 95% CI: 0.85–2.62) [8]. Additionally, we conducted a meta-analysis to investigate the impact of pulmonary complications after esophagectomy on survival [10]. Patients with pulmonary complications had significantly worse five-year OS (HR: 1.37, 95% CI: 1.16–1.62, *p* = 0.0003), five-year cancer-specific survival (CSS) (HR: 1.60, 95% CI: 1.35–1.89, *p* < 0.00001), and five-year disease-free survival (DFS) (HR: 1.18, 95% CI: 1.00–1.38, *p* = 0.04).

### 2.2. Anastomotic Leakage

Using information recorded between 2011 and 2012 from a nationwide database in Japan, we reported that the anastomotic leakage rate after esophagectomy was 12.6% (1203/9584) [5]. Additionally, Markar et al., reported that using a multicenter database in France, postoperative severe anastomotic leakage (8.5%, 208/2439) negatively affected long-term survival significantly [15].

In contrast, we previously reported that, in a single institution, anastomotic leakage after esophagectomy (19.4%, 55/284) did not affect OS [4]. Furthermore, we analyzed data from the JCOG9907 trial; OS of patients with anastomotic leakage (13.8%, 21/152) was nearly identical to that of patients without leakage (HR: 1.06, 95% CI: 0.52–2.13); PFS showed the same tendency (HR: 1.28, 95% CI: 0.71–2.32) [8]. However, we conducted a meta-analysis to investigate the impact of anastomotic leakage after esophagectomy on survival and reported that patients with anastomotic leakage had significantly worse five-year OS (HR: 1.18, 95% CI: 1.04–1.33, *p* = 0.01), five-year CSS (HR: 1.81, 95% CI: 1.11–2.95, *p* = 0.02), and five-year DFS (HR: 1.13, 95% CI: 1.03–1.25, *p* = 0.01) [10].

### 2.3. Overall Complications

Using information recorded between 2011 and 2012 from a nationwide database in Japan, we reported that the rate of overall morbidity after esophagectomy was 42.8% (4102/9584) [5]. Ancona et al., and Ferri et al., reported that overall postoperative complications did not affect long-term survival (16.3%, 85/522 and 22.6%, 98/434, respectively) [12,16]. However, Baba et al., and Saeki et al., recently reported that overall postoperative complications negatively affected long-term survival (43.2%, 217/502 and 26.6%, 154/580, respectively) [13,14].

We conducted a meta-analysis to investigate the impact of overall morbidity after esophagectomy on survival and reported that the overall postoperative morbidity had significantly worse five-year OS (HR: 1.16, 95% CI: 1.06–1.26, *p* = 0.001) and five-year CSS (HR: 1.28, 95% CI: 1.11–1.48, *p* = 0.0009) [10].

It was possible that the worsening of the general condition after postoperative complications lead to a delay or cessation of additional therapy after esophagectomy and led to esophageal cancer recurrence [4].

## 3. Clinical Significance of Proinflammatory Cytokines

Persistent infection or chronic inflammation significantly contributes to tumorigenesis and tumor progression. C-X-C motif ligand 8 (CXCL8) is a chemokine that acts as an important multifunctional cytokine to modulate tumor proliferation, invasion, and migration in an autocrine or paracrine manner [17]. CXCL8 and its cognate receptors, C-X-C chemokine receptor 1 (CXCR1) and C-X-C chemokine receptor 2 (CXCR2), may mediate the initiation and development of various cancers, including breast cancer [18], prostate cancer [19], lung cancer [20], colorectal carcinoma [21], and melanoma [22]. Further, CXCL8 integrates with multiple intracellular signaling pathways to produce coordinated effects. Additionally, neovascularization, which provides a basis for fostering tumor growth and metastasis, is now recognized as a critical function of CXCL8 in the tumor microenvironment [17].

The complication-specific factors that negatively affected long-term survival included pulmonary complications, involving a generalized infection that produced strong impairment of the immunological system leading to esophageal cancer recurrence [4]. Furthermore, we previously reported that infectious postoperative esophagectomy complications significantly increased the levels of inflammatory cytokines, such as CXCL6 and CXCL8 [23]. Increased expression of CXCL8 and its receptor, CXCR2, has been correlated with tumor progression after esophagectomy [24,25]. Thus, pulmonary complications may be related to tumor progression by promoting inflammatory cytokines, such as CXCL8, which negatively affects CSS and DFS [4]. Additionally, anastomotic leakage could result in the spread of viable tumor cells locally from stapled or sutured anastomoses. Locoregional recurrence after anastomotic leakage could be related to a proinflammatory response that promotes tumor growth [15]. Pulmonary infectious complications and anastomotic leakage have been related to tumor progression by developing inflammatory cytokines, such as CXCL8 [10]. Moreover, anastomotic leakage after esophagectomy have been shown to negatively affect CSS and DFS.

## 4. The Major Signaling Pathways of CXCL8 in Esophageal Cancers

**Figure 1:**
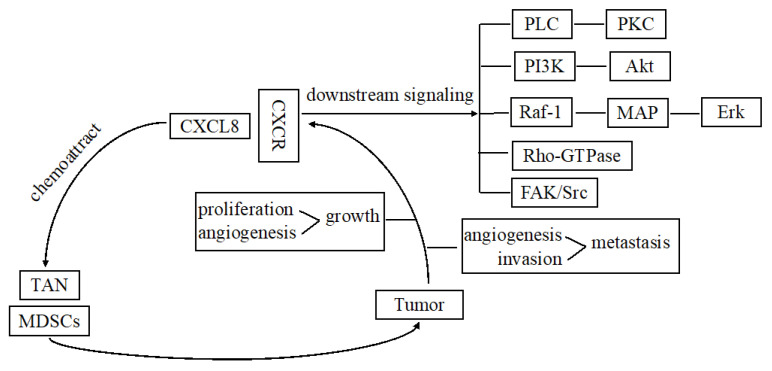
Diagram summarizing the major signaling pathways of CXCL8 in cancers.

CXCL8 chemoattractant myeloid-derived suppressor cells and tumor-associated neutrophils in the tumor microenvironment are associated with immune suppression [17]. At the cellular level, CXCL8 binds to G-protein-coupled receptors, namely, CXCR1 or CXCR2, resulting in G-protein activation. Heterotrimeric Gα and βγ subunits stimulate the main effectors PLC and PI3K to induce phosphorylation of PKC and Akt, respectively. The two signaling pathways activate respective transcription factors associated with survival, angiogenesis, and migration of tumor cells. Additionally, CXCL8 activates nonreceptor tyrosine kinases (e.g., Src and FAK) and Rho-GTPase family members, which promote proliferation, survival, motility, and invasion of cells. The activated Raf-1/MAP/Erk signaling cascade contributes to the proliferation and survival of cells (Figure 1).

The global gene expression analysis suggests that CXCL8/CXCR2 signaling reduces the expression of SFRP1, an antagonist of the Wnt signaling pathway [25]. SFRP1 expression has been demonstrated in human esophageal mucosa, especially in the lamina propria and basal cells. Additionally, Wnt/Frizzled signaling plays an essential role in embryonic development, cell differentiation, and cell proliferation [26]. SFRP1 promoter hypermethylation has been observed in esophageal squamous cell carcinoma (ESCC) and esophageal adenocarcinoma [27,28]. Thus, the silencing of the SFRP1 gene may be a mechanism by which CXCL8/CXCR2 signaling enhances cell proliferation in ESCC.

## 5. Multidisciplinary Team Management for Prevention of Postoperative Complications after Esophagectomy

In April 2017, we launched the multidisciplinary Hamamatsu Perioperative Care Team (HOPE) for all surgical patients [29]. Additionally, we developed a reinforced intervention strategy, particularly for esophagectomy. HOPE consisted of surgeons, nurses, rehabilitation physicians, physiotherapists, speech-language-hearing therapists, dieticians, and pharmacists who collaborated with the nutritional support, infection control, and palliative care teams (Table 1). By introducing HOPE, we supported the patient′s perioperative period from various aspects. Overall, 125 patients underwent esophagectomy and gastric conduit reconstruction for esophageal or esophagogastric junction cancer between January 2014 and December 2018 at the Department of Surgery in Hamamatsu University School of Medicine. The patients were categorized into the pre-HOPE group, including 62 patients who underwent esophagectomy before the formation of HOPE, and the HOPE group, including 63 patients who underwent esophagectomy after introducing HOPE. The incidence rates of postoperative pneumonia after esophagectomy (Clavien–Dindo classification grade 2 or higher) were significantly lower in the HOPE group (14%, 9/63) than in the pre-HOPE (29%, 18/62) group (*p* = 0.037) [29]. The introduction of the multidisciplinary HOPE was associated with a significant reduction in postoperative pneumonia incidence, leading to improved long-term survival after esophagectomy [29]. The mean body weight at 1, 3, 6, and 12 months postoperatively indicated that the weight loss in the HOPE group was significantly less than that in the pre-HOPE group (*p* < 0.001) [29]. Moreover, the loss of postoperative psoas muscle index was significantly lower in the HOPE group than the pre-HOPE group (92.2% ± 21.3% vs. 74.8% ± 21.9%, *p* < 0.001) [29].

However, Valkenet et al., recently reported from a multicenter randomized controlled trial that preoperative inspiratory muscle training did not decrease the rate of pneumonia after esophagectomy [30]. Not all patients might require preoperative inspiratory muscle training, thanks to the development of minimally invasive esophagectomy. In the future, it would be important to determine patients who need preoperative inspiratory muscle training and offer intensive intervention for these patients.

## 6. Introduction of Minimally Invasive Esophagectomy

Minimally invasive esophagectomy (MIE) has been performed more often to treat esophageal cancer since it was first detailed in 1992 [31]. According to a Japanese national database, the ratio increases annually, including 66.8% (4209/6298) patients who underwent video-assisted thoracoscopic surgery (VATS) for esophageal cancer in 2019 [32]. According to an analysis performed by the Esophageal Complications Consensus Group, the most common complications of MIE are pneumonia, arrhythmia, anastomotic leakage, conduit necrosis, chylothorax, and recurrent laryngeal nerve palsy [33]. Several studies comparing the short-term outcomes of VATS and open esophagectomy (OE) based on nationwide or prospective data have been recently published [34]. Those studies reported that the overall rate of surgical complications was higher for VATS than for OE, although VATS was associated with lower rates of respiratory complications than OE [34]. Robot assistance provides an enlarged, three-dimensional view field and improves the surgeon’s dexterity owing to surgical wrists and tremor filtration [35]. We recently reported that robot-assisted minimally invasive esophagectomy (RAMIE) reduced overall postoperative complications after esophagectomy compared with OE and VATS (OE; 77/110(70.0%), VATS; 93/127(73.2%), RAMIE; 11/35(31.4%), *p* < 0.001, respectively) [36]. RAMIE could reduce postoperative complications, including infectious complications, improving long-term survival after esophagectomy [36].

Additionally, based on a randomized phase three controlled trial, Mariette et al., reported that laparoscopic gastric mobilization significantly reduced the postoperative pulmonary complications (18%, 18/102) compared with open gastric mobilization (30%, 31/103) after open thoracic esophagectomy (odds ratio (OR): 0.50, 95% CI: 0.26–0.96, *p* < 0.001) [37]. It was also reported that at 3 years, OS was 67% (95% CI, 57–75) in the laparoscopic gastric mobilization group compared with 55% (95% CI, 45–64) in the open gastric mobilization group; DFS was 57% (95% CI, 47–66) and 48% (95% CI, 38–57) [37]. Moreover, we conducted a meta-analysis, including a randomized controlled trial that assessed the ability of laparoscopic gastric mobilization to prevent postoperative complications after open thoracotomy or thoracoscopic esophagectomy [38]. Laparoscopic gastric mobilization performed after open thoracotomy resulted in a significant reduction in postoperative pulmonary complications (OR: 0.47, 95% CI: 0.27–0.82, *p* = 0.008) and postoperative mortality (OR: 0.49, 95% CI: 0.25–0.94, *p* = 0.03) [38]. Similarly, laparoscopic gastric mobilization after thoracoscopic esophagectomy resulted in significantly reduced postoperative pulmonary complications (OR: 0.56, 95% CI: 0.37–0.84, *p* = 0.005) and anastomotic leakage (OR: 0.59, 95% CI: 0.39–0.91, *p* = 0.02) [38]. We concluded in the meta-analysis that laparoscopic gastric mobilization may be recommended for reducing postoperative pulmonary complications after esophagectomy, irrespective of the thoracic approach. Thus, laparoscopic gastric mobilization could improve long-term survival after esophagectomy.

## 7. Definitive Chemoradiotherapy

Esophagectomy is a highly invasive procedure associated with a high risk of postoperative complications; therefore, definitive chemoradiotherapy (dCRT) is a treatment alternative when esophagectomy is contraindicated. dCRT is the standard treatment in cStage I/II/III ESCC with equivalent OS, including salvage treatment compared with esophagectomy [39,40].

We recently reported no significant difference in OS between dCRT and radical esophagectomy in patients with cStage I ESCC [41]. Furthermore, in subgroup analyses, we previously reported that pneumonia significantly negatively affected OS for patients aged >65 years or for those in cStage I. However, pneumonia did not significantly impact the OS of patients aged <64 years or those in cStage II/III/IV [4]. Additionally, smoking history was strongly correlated with the development of pneumonia [4]. Thus, the results suggested that patients aged >65 years or those in cStage I were unsuitable for esophagectomy if they were at high risk of esophagectomy, such as with smoking history, and these patients might be recommended dCRT as a treatment option instead of esophagectomy.

## 8. Conclusions

Postoperative infectious complications after esophagectomy have been related to tumor progression by developing inflammatory cytokines, such as CXCL8. To prevent postoperative complications after esophagectomy, the introduction of MIE and multidisciplinary team management would be effective. Furthermore, tailor-made treatments, such as avoiding radical esophagectomy and performing dCRT, could become necessary for patients with high perioperative risk, such as the elderly.

## Figures and Tables

**Table 1:** HOPE program.

Dental Screening and Professional Cleaning
Cessation of smoking and drinking
Measurement of physical fitness
Respiratory exercise using a device
Nutritional screening and support
Sufficient pain control
Early ambulation
Early enteral nutrition via jejunostomy tube
Swallowing evaluation

## Data Availability

No new data were created or analyzed in this study. Data sharing is not applicable to this article.
